# Modeling open surgery in mice to explore peritoneal damage, carbon dioxide humidification and desmoidogenesis

**DOI:** 10.1515/pp-2019-0023

**Published:** 2019-11-02

**Authors:** Timothy Chittleborough, Shienny Sampurno, Sandra Carpinteri, Andrew Craig Lynch, Alexander Graham Heriot, Robert George Ramsay

**Affiliations:** GI Cancer Program, Peter MacCallum Cancer Centre, 305 Grattan Street, Melbourne 3000, Victoria, Australia; Surgery, Peter MacCallum Cancer Centre, Melbourne, Victoria, Australia

**Keywords:** desmoid tumors, laparotomy, mouse surgery, peritoneum, wound healing

## Abstract

**Background:**

The exposure of the peritoneum to desiccation during surgery generates lasting damage to the mesothelial lining which impacts inflammation and tissue repair. We have previously explored open abdominal surgery in mice subjected to passive airflow however, operating theatres employ active airflow. Therefore, we sought an engineering solution to recapitulate the active airflow in mice. Similarly, to the passive airflow studies we investigated the influence of humidified-warm carbon dioxide (CO_2_) on this damage in the context of active airflow. Additionally, we addressed the controversial role of surgery in exacerbating desmoidogenesis in a mouse model of familial adenomatous polyposis.

**Methods:**

An active airflow mouse-operating module manufactured to produce the equivalent downdraft airflow to that of a modern operating theatre was employed. We quantified mesothelial cell integrity by scanning electron microscopy (SEM) sampled from the peritoneal wall that was subjected to mechanical damage or not, with and without the delivery of humidified-warm CO_2_. To explore the role of open and laparoscopic surgery in the process of desmoidogenesis we crossed *Apc^min/^*^+^ C57Bl/6 mice with *p53*^+/−^ mice to generate animals that developed desmoid tumors with 100% penetrance.

**Results:**

One hour of active airflow generates substantial damage to peritoneal mesothelial cells and their microvilli as measured at 24 h post intervention, which is significantly greater than that generated by passive airflow. Use of humidified-warm CO_2_ mostly protects the mesothelium that had not experienced additional mechanical (surgical) damage at 24 h. Maximal damage was evident in all treatment groups regardless of flow or use of gas. At day 10 mechanically-damaged peritoneum remains in mice but is essentially repaired in the gas-treated groups. Regarding desmoidogenesis, operating procedures did not increase the frequency of desmoid tumors but their frequency correlated with time following surgery but not age of mice.

**Conclusions:**

Active airflow generates more peritoneal damage than passive airflow and is reduced significantly by the use of humidified-warm CO_2_. Introduced peritoneal damage is largely repaired in mice by day 10 with gas. Desmoid tumor incidence is not increased substantially by surgery itself but rises over time following surgery compared to non-surgery mice.

## Introduction

Surgery employing laparotomy involves exposure of the abdomen and its associated visceral and peritoneal tissue surfaces to the risk of surface desiccation. This exposure is not passive as the modern operating theatre employs active filtered air exchange of approximately 20 room volumes per hour [1] leading to desiccation [2] and heat loss [3]. The visceral and parietal peritoneal surfaces are covered by an integral layer of mesothelial cells augmented by a lawn of apical microvilli [4]. By using mouse models, such desiccation is readily evident and measureable by documenting changes to the cellular architecture [5]. Such damage to the peritoneum exacerbated the propensity of cancer cells to implant with the potential to initiate peritoneal carcinomatosis [6].

In an attempt to address and to minimize open surgery-associated peritoneal damage, we have explored the use of humidified-warm carbon dioxide (HWCO_2_) delivered via a bespoke mini gas perfusion device. In this passive airflow model, we reported protection of the peritoneal surfaces by using HWCO_2_ [5], similar to that employed in human open surgery [7]. Here, we advanced our investigation of peritoneal damage by engineering a surgery theatre set-up designed for mice whereby airflow was instead delivered by active airflow. The consequence of greater airflow was increased peritoneal damage compared to passive airflow yet this exacerbated damage was minimized by co-delivery of HWCO_2_.

Previously, we investigated the impact of peritoneal damage on tumor cell implantation where exogenous cancer cells were introduced into the peritoneal cavity [5, 6]. To examine a different aspect of surgery impact on tumorigenesis here we used a genetic model (*Apc^min/^*^+^*:p53KO*) predisposed to endogenous abdominal wall desmoid tumors, desmoidogenesis [8, 9]. This model was of interest because abdominal surgery significantly increases the risk of development of this neoplasm in patients with familial adenomatous polyposis (FAP) [10, 11, 12, 13, 14]. Furthermore, desmoid tumors cause serious morbidity and mortality in patients with FAP, particularly following prophylactic colectomy [10, 14]. Accordingly, we used four approaches: open and laparoscopic interventions, with or without HWCO_2_ to address the consequences of these different surgical methods on desmoidogenesis.

## Materials and methods

### Mouse surgery studies

**Figure 1:**
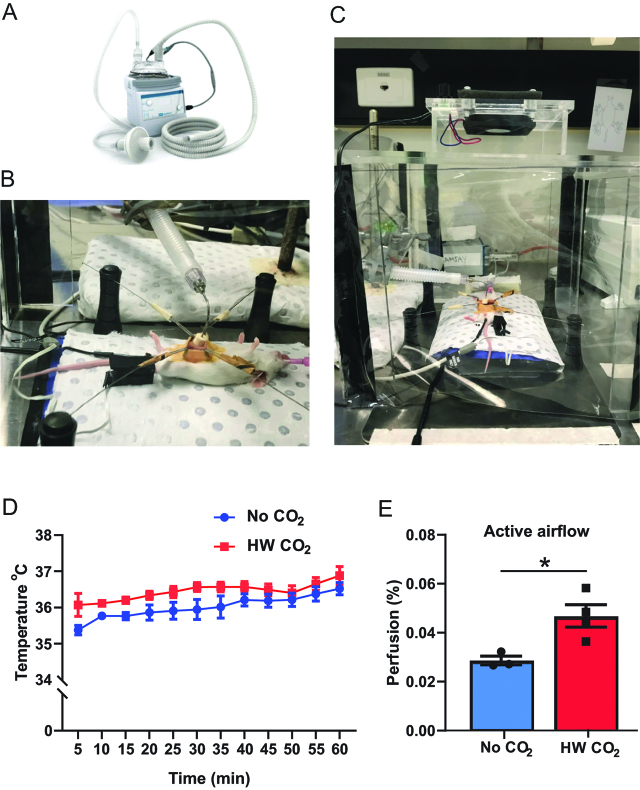
Mouse surgery set up, engineered devices and effect of humidified-warm CO_2._

Female BALB/c mice aged 7–12 weeks old, weighing 17–25 g were used according to the Institutional Animal Ethics Committee approval and the National Health and Medical Research Council of Australia as previously described [5]. Mice were purchased from the ARC (Perth, Australia) and housed in the PMCC facility with a 12 h light-dark cycle allowing *ad libitum* access to food and water. The delivery of HWCO_2_ was via a HumiGard (Fisher and Paykel Healthcare, New Zealand) recalibrated to produce a pressure of 2 mmHg appropriate to mice (Figure 1(a)).

(A) Commercial humidified-warm CO_2_ (HWCO_2_) apparatus reprogrammed to deliver equivalent gas flows to recapitulate that used in patients. Anesthetized mouse showing insufflation ports and intubation tubing. (B) Anesthetized mouse subjected to laparotomy with CO_2_ delivery device and perfusion measuring clamp. (C) Engineered mouse operating suite with forced down flow of air to replicate operating theatre conditions. (D) Temperature monitoring of mice during a laparotomy procedure with active airflow delivered by (C) with or without provision of HWCO_2_ via (B). (E) Perfusion of oxygen to the right rear paw measured in mice during a 1-h laparotomy procedure with active airflow (*p<0.05, two-tailed t-test; mean±SEM; n=3 mice per group).

Normal BALB/c mice were anesthetized, intubated and placed on a warming pad as for insufflated mice used in previous laparoscopic studies [6] with or without a miniaturized gas diffuser designed for open surgery studies (Figure 1(b)). For these laparotomy investigations, mice were randomized to two groups: (i) open surgery with exposure to active airflow and (ii) open surgery with active airflow plus H-WCO_2_ diffusion using a purpose engineered operating suite (Figure 1(c)). Replicating surgical trauma on the peritoneal abdominal wall has been described [5]. Briefly, peritoneal wall damage was simulated using sterile plastic serrated forceps on one side of the peritoneum. This was performed by a sole investigator whereby the mini gas diffuser was removed and the sterile plastic forceps used to abrade the mesothelial layer with five strokes to the right-sided wall at the 30 min time point standardized to the middle of the peritoneum approximately 1 cm from the abdominal incision. Peritoneal wall from the left side was used as a non-surgical intervention control. C57BL/6 mice (see further details below) were similarly managed to above but were ventilated with a nose cone (instead of intubated) due to the small size of the *Apc^min/^*^+^:*p53*^+/−^ *mice.*

### Equipment

To establish operating room conditions that accurately replicate that employed in the operating theatre a custom miniature laminar flow system was developed. Laminar flow was created with a perforated ceiling and a computer-cooling fan mounted directly above the mouse operating space. Flow velocity tested with a hot wire anemometer and calibrated to be 0.5 m/sec in accordance with the DIN standard 1946 for hospital ventilation. Fisher and Paykel Healthcare engineered an open-sided box constructed from Perspex with the aim of positioning the fan over the anesthetized mouse. Thus, the “mouse” operating suite recapitulated the environment used for patients (Figure 1(c)). Mouse vital signs were monitored as previously described as well as tissue analyses [5, 6]. Briefly, for perfusion indices presented in this study these were determined using an inbuilt pulse oximeter and SpO_2_ sensor (PhysioSuite™; Kent Scientific, Torrington, CT, USA) which analyzes the rate of change between oxygenated hemoglobin and reduced hemoglobin as arterial oxygen saturation expressed as a percentage.

### Desmoidogenesis studies

A model of desmoidogenesis was developed by crossing of *Apc^Min/^*^+^ mice [15, 16] with *p53*^+/−^ mice on a C57Bl/6 background based upon a similar model using a different *Apc* mutation reported previously [9]. Due to breeding constraints due to reduced viability of female *p53*^−/−^ mice [17] only male mice were evaluated. Genotyping by polymerase chain reactions for the *p53* exon deletion used the following primers: p53x6s 5ʹ-TTATGAGCCACCCGAGGT-3ʹ; p53x7as 5ʹ-TATACTCAGAGCCGGCCT-3ʹ; neo18.5 5ʹ-TCCTCGTGCTTTACGGTATC-3ʹ, reaction ratio 1:1:2 to generate product of 600 bp (KO) and 450 bp (WT), respectively. For the *Apc^min^* mutation detection PCRs employed the following primers: MAPCMT 5ʹ-TGAGAAAGACAGAAGTTA-3ʹ; MAPC15 5ʹ-TTCCACTTTGGCATAAGG-3ʹ; MAPC9 5ʹ-GCCATCCCTTCACGTTAG-3ʹ to generate 600 bp (WT) and 300 bp (min allele) products, respectively. In these studies, no tissue was removed and the only intervention was either laparoscopy or laparotomy in order to determine the impact of surgical approach on subsequent desmoid risk. Desmoid burden was quantified macroscopically (number of lesions present) as well as microscopically at sites of surgical trauma (i. e. laparotomy wound).

### Semi-quantitative analyses

Mice were culled at 24 h and 10 days. Scanning electron microscopy (SEM) was used to evaluate changes to morphology and alterations quantified using a customary scale adapted from the H-score method. Essentially, microvilli were evaluated per cell as normal, damaged or shortened and absent. The proportions were expressed as a percentage of the total. For mesothelial cells, integrity cells were scored as normal, retracted or bulging expressed as a percentage of the total [6].

### Statistical analyses

Data are expressed as mean±standard error of the mean evaluated using GraphPad^®^ Prism 6 (La Jolla, CA, USA) and analyzed using 1- or 2-way ANOVA with Tukey’s multiple comparisons test or two-tailed unpaired t-test. A p-value of less than 0.05 was considered statistically significant.

## Results

### Design of animal operating suite

Laminar flow ventilation is standard in current day operating rooms. Investigations have demonstrated the efficacy of the system to reduce the number of particles and bacteria in the operating room air. To achieve effective laminar flow, highly filtered air is blown with uniform velocity in single parallel flow lines over the target area. The air may be “changed” more than 300 times per hour at a flow velocity of 0.2–0.5 m/s. This continual flow is a relevant variable when recapitulating the operating environment in animal models of open abdominal surgery as it may impact the local temperature of the wound and the evaporative capacity of the operating environment has a desiccating effect on abdominal surfaces [5, 18].

To explore different modes of open surgery for testing the effects of HWCO_2_ on the peritoneum mice were subjected to intubation and anesthesia as previously described [6] with an option to deliver HWCO_2_ via a proprietary humidification device shown in Figure 1(a). A laparotomy model was developed where mice were exposed to ambient and passive airflow with and without provision of HWCO_2_ delivered via a gas diffusion device (Figure 1(b)) [5]. To recapitulate active airflow indicative of a modern operating theatre a Perspex^®^ box structure was engineered to provide a constant down draft of ambient air to mice undergoing laparotomy (Figure 1(c)) with or without the gas diffusor device.

### Temperature maintenance under active airflow

As all mice were the same strain (BALB/c) and balanced for gender the effects of various surgery modalities could be robustly compared within new, and against previously published data [5]. A rectal probe was employed to as a reliable method of recording core body temperature over time [19]. The current study focused on the active airflow surgery model finding that HWCO_2_ allows the maintenance of statistically higher stable core body temperature for the first 15 min for each 5-min interval from the beginning and overall for all time points of the procedure compared to active airflow alone (Figure 1(d)). However, the temperatures measured previously in mice undergoing laparoscopy [6] and laparotomy [5] were all higher by approximately 1 °C (37.5 °C) where HWCO_2_ was used. These data indicate that the active flow of air into an open abdomen is most effective at lowering core body temperature in mice.

### Total body perfusion under active airflow

We monitored vital signs in all mice over the procedure and collected perfusion at the distant hind paw as previously described [5] to find that HWCO_2_ allowed for significantly increased perfusion compared to active airflow alone (Figure 1(e)). The degree of perfusion was reduced by approximately one-third when compared to passive flow data but comparable to the passive flow with additional HWCO_2_ [5].

### Measuring peritoneal integrity after laparotomy under active airflow

**Figure 2:**
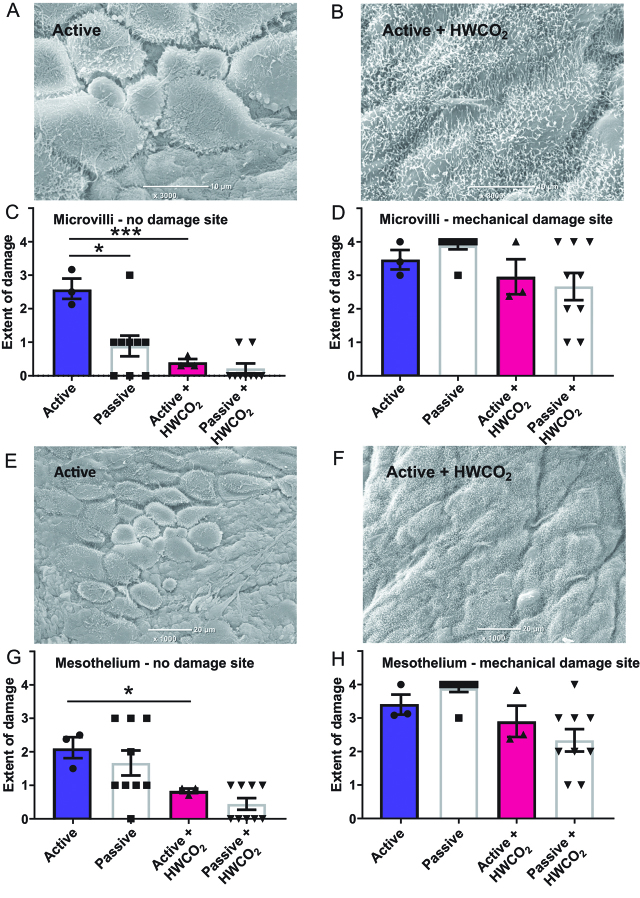
Comparing peritoneal damage following laparotomy at 24 h post-treatment.

At 24 h, post-surgery SEM micrographs reveal extensive loss of microvilli under active flow conditions (Figure 2(a)) while microvillus integrity was essentially comparable to baseline when HWCO_2_ was employed (Figure 2(b)). The quantitative measures of extent of damage are presented in Figure 2(c). Historical data from passive airflow work [5] have been included to emphasize the effects of active vs. passive flow. Although this previous study by definition was conducted at an earlier time the strain of mouse, age range, gender mix and experimental team were comparable. When mechanical damage was introduced to one side of the peritoneal wall and evaluated 24 h later all groups displayed maximal microvilli damage (Figure 2(d)).

(A&B) Scanning electron micrographs (SEM) showing microvilli under active airflow without and with HWCO_2_. (C&D) Quantification of microvilli integrity/damage in mice subjected to active airflow compared to passive airflow without or with HWCO_2_. Mechanical damage introduced to the opposite wall of the peritoneum evaluated under the four treatment conditions. (E&F) SEM showing mesothelial cells under active airflow without and with HWCO_2_. (G&H) Quantification of mesothelial cell integrity in mice subjected to active airflow compared to passive airflow without or with HWCO_2_. Mechanical damage introduced to the opposite wall of the peritoneum evaluated under the four treatment conditions (*p<0.05, ***p=0.001; two-tailed t-test; mean±SEM; n=3−8 mice per group; size bars 10  μm and 20  μm).

Figure 2(a) and 2(e) highlights the separation of cells from each other across the mesothelial cell sheet that typically lines the peritoneum. This effect was most pronounced in the active airflow group and the delaminating/retraction of cell was reduced when HWCO_2_ was used (Figure 2(f) and (g)). By contrast, the extent of mesothelial damage was pervasive in all groups when mechanical damage was assessed (Figure 2(h)).

### Longer term measures of peritoneal integrity

**Figure 3:**
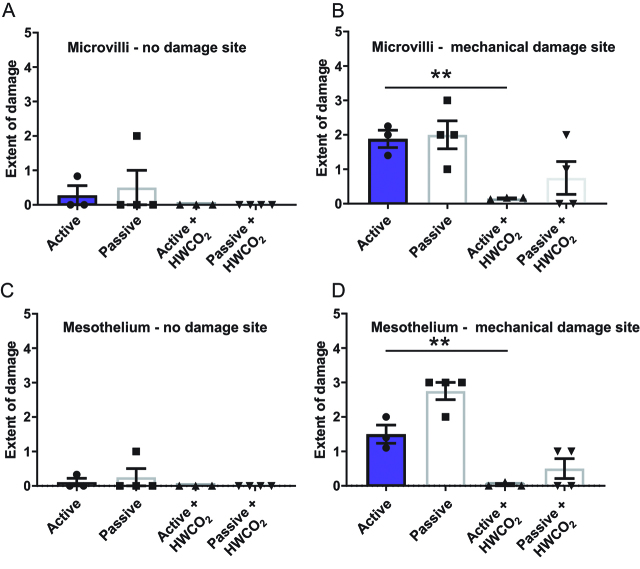
Comparing peritoneal damage following laparotomy at 10-day post-treatment.

By day 10, microvilli and mesothelial cell integrity is essentially normal in all groups of mice regardless of HWCO_2_ delivery (Figure 3(a) and (c)). However, as previously discovered the introduction of mechanical damage at the time of surgery has an enduring impact on microvilli restoration and mesothelial cell integrity (Figure 3(b) and 3(d)). By both cellular indicators, the use of HWCO_2_ appears to accelerate or perhaps allow tissue repair such that base line normal cellular SEM micrographs were observed at day 10. These data suggest that HWCO_2_ provides protection to both exposed and additionally mechanically damaged peritoneum over the 1 h of operating time.

(A&B) SEM evaluation of microvilli integrity under active and passive airflow with or without HWCO_2_. (C&D) SEM evaluation of mesothelial cell integrity under active and passive airflow with and without HWCO_2_ (**p<0.01; two-tailed t-test; mean±SEM; n=3−4 mice per group).

### Development of a desmoid tumor model in mice

**Figure 4:**
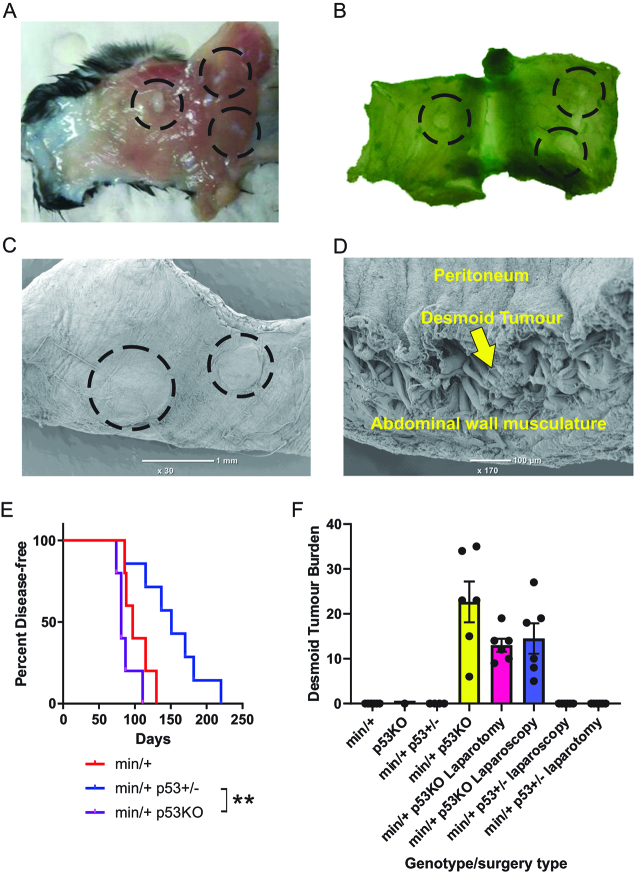
Development of a mouse model for the study of the effects of surgery on desmoid tumorigenesis.

To generate a model of desmoidogenesis *Apc^Min/^:p53*^−/−^ mice were bred on a C57BL/6 background. The mouse model allowed one hundred percent penetrance of desmoid tumors. Mice were culled due to illness that most typically involved anemia associated with bloody stools or morbid behavior. This pathology is typical of *Apc^Min/^*^+^ mice that have developed intestinal adenomas which were subsequently confirmed by examination of the large and small intestines. Co-incident with adenoma formation were the development of desmoid tumors on the abdominal walls of *Apc^Min/^:p53*^−/−^ (Figure 4(a)) but not *Apc^Min/^:p53*^+/−^ or *Apc^Min/^*^+^ mice. Such tumors are highlighted by staining fresh specimens with Fast Green (Figure 4(b)) and were further characterized by scanning electron microscopy (Figure 4(c) and 4(d)). Clinical features of mice evaluated in this study are reported in Table 1.

(A) Peritoneal wall highlighting desmoid tumors (broken-line circles) in *min/+ p53KO* mice. (B) Contrast staining used to enumerate desmoid tumor burden in mice. (C&D) Low (scale bar=1 mm) and high magnification (scale bar=100  μm) SEM micrographs showing high-resolution desmoid tumor morphology (yellow arrow). (E) Percent-disease-free survival in mice of different genotypes. (F) Desmoid tumor burden in *Apc^min/^*^+^*:p53*^−/−^ (*min:p53KO*) mice subjected to laparoscopic or laparotomy-based surgery (**p<0.01; two-tailed t-test; mean±SEM; n=4−6 mice per group).

**Table 1:** Characteristic of clinical features and desmoid tumor burden in *Apc^min/^*^+^*:p53*^−/−^ mice.

*Apc^min/^*^+^*:p53*^−/−^	Age at surgery	Operative findings	Reason for culling	Age at death	Days post-surgery at death	Desmoid tumor burden	Adenoma burden (small, large bowel)	Other findings
1: Laparoscopy, dry-cold CO_2_	52	N/A	Anemia	76	24	5.	0, 3	Mesenteric mass – pancreatic acinar cell carcinoma
	57	N/A	Anemia	112	55	18	14, 1	Mesenteric mass – lymphoproliferative
	43	N/A	Enlarging neck mass	77	34	8	4, 3	Enlarging neck mass* *~1.5 cm in size – sarcomatous. Mesenteric mass – lymphoproliferative
2: Laparoscopy, humidified-warm CO_2_	53	N/A	Anemia	113	60	10	17, 3	Intussusception. Lesion on hind leg muscle – peripheral desmoid
	42	N/A	Anemia	97	55	27	16, 4	Mesenteric mass – lymphoproliferative
	47	N/A	Enlarging LIF mass	103	56	19	11, 1	5 Retroperitoneal desmoids. LIF mass – angiosarcoma
3: Laparotomy, conventional	56	10 desmoids	Prolapse	72	16	10.	3, 6	Pancreatic nodule – pancreatic acinar cell carcinoma
	54	10 desmoids	Tachypnea	99	45	14	19, 0	Grossly enlarged thymus – lymphoma; 2 retroperitoneal desmoids
	46	15 desmoids	Anemia	109	63	14	12, 3	Mesenteric mass – lymphoproliferative, multiple intussusceptions, 6 retroperitoneal desmoids
4: Laparotomy, humidified-warm CO_2_	48	13 desmoids	Anemia	96	48	12	19, 2	3 retroperitoneal desmoids. Mesenteric mass – lymphoproliferative
	47	7 desmoids	Anemia	85	38	9	16, 2	Mesenteric mass – lymphoproliferative
	44	9 desmoids	Anemia	105	61	19	11, 4	Grossly enlarged thymus – lymphoma

*Apc^Min/^*^+^:p53^−/−^ mice had a slightly reduced time to development of disease compared to *Apc^Min/^*^+^ mice while the *p53*^+/−^ genotype afforded extended disease-free survival (Figure 4(e)) and more rapidly than expected for mice on a *p53*^+/−^ or *p53*^−/−^ background alone [17]. The small and large intestinal adenomas were observed in all mice with the mutant *Apc* allele. However, only *p53*^−/−^ genotypes had the additional burden of desmoid tumors (Figure 4(f)). Of note only *p53*^−/−^ male mice were investigated in these studies due to the general low yield of *p53*^−/−^ female mice. This sex bias presents a limitation of this pre-clinical study because desmoid tumors are twice as frequent in females vs. males [20].

### Effect of surgery approach on desmoid tumor formation

To evaluate the effect of laparoscopic and laparotomy surgery on the degree of desmoid formation in *Apc^Min/^*^+^*:p53*^−/−^ mice animals were culled when declared by an independent animal technician blinded to the genotype and surgery approach. Similar tumor burdens were found in the no-intervention and surgery groups (Figure 4(f)).

**Figure 5:**
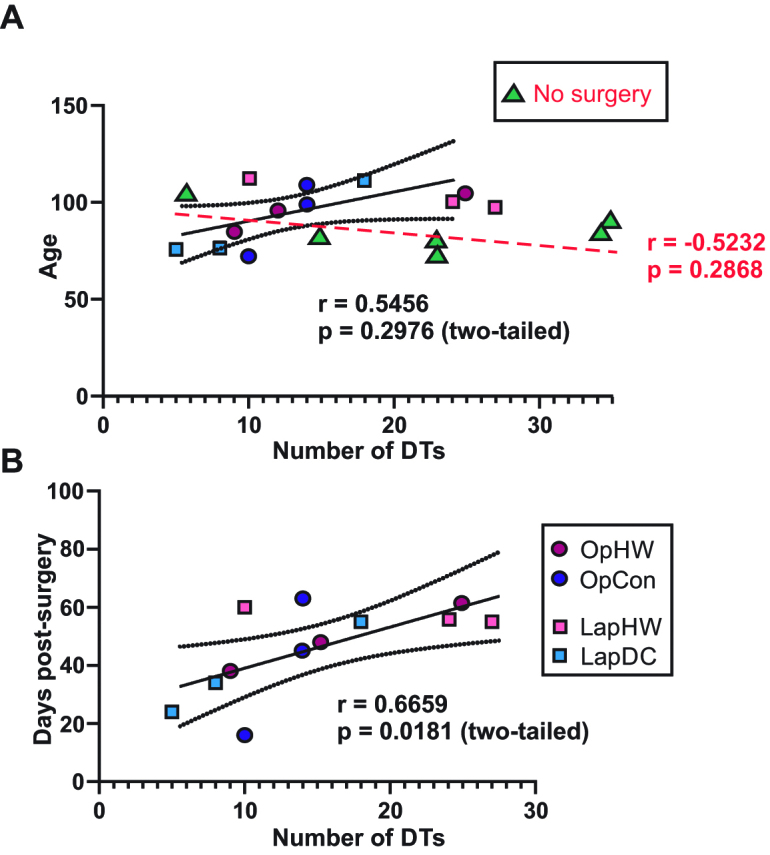
Desmoid tumor burden increases with time following surgery but not age.

The humidified-warm (HW) and dry-cold (DC) CO_2_ groups were pooled according to surgery approach whereby there were no significant relationship between desmoid tumor burden and age at cull (Figure 5(a)). However, when the desmoid tumors were evaluated in terms of days post-surgery there was a trend toward an increased burden in relation to time following surgery. By contrast, adenoma burden in *Apc^Min/^*^+^*:p53*^−/−^ mice increased with age as expected as well as with time from surgery (Supplemental Figure 1). There was no significant relationship between adenoma tumor burden, CO_2_ type or surgery modality although there was perhaps a trend toward more adenomas in mice that received HWCO_2_ but these mice were generally older at the time of cull consistent with an increase in adenomatogenesis with age in the *Apc^min/^*^+^ model. When all genotypes were considered collectively vs. HWCO_2_ and either DCCO_2_ or passive airflow there was no significant differences in time to cull (data not shown).

(A&B) Number of desmoid tumors are greater in all *Apc^min/^*^+^*:p53KO* mice with or without surgery (green triangles) at age of cull or days post-surgery (OpHW – laparotomy with HWCO_2_; OpActive – laparotomy with active airflow; LapHW – laparoscopy with HWCO_2_; Lap – laparotomy conventional airflow). Linear regression and line of best fit are shown for no surgery (red line).

## Discussion

To explore whether there might be a role for HWCO_2_ in peritoneal tissue protection we have developed a series of mouse surgery platforms that cover laparoscopic [6] and open surgery [5]. Mice are not perfect for the modeling of human pathology but the ultrastructure, cell biology and gene expression profiles of mouse and human peritoneal tissues are very similar [21]. However, individual mouse to mouse, particularly on a strain-by-strain basis, are genetically and phenotypically uniform allowing for studies with tight experimental design.

Our previous open surgery studies employed passive airflow where HWCO_2_ led to measurable and significant tissue protection of the peritoneum [5]. Here we added the sophistication of active airflow to model the effects of the active airflow indicative of the human operating suite. This change to active airflow increased the degree of damage compared to passive airflow. Similarly, HWCO_2_ delivered into the abdominal cavity during open surgery allowed maintenance of normothermia and oxygen perfusion in mice over the 1-h period of the study.

At 24 h post-surgery active airflow by all measures showed increased tissue damage at the cellular and subcellular level well above base line. This was most notably when microvilli integrity was evaluated. HWCO_2_ use reduced this damage as well as that observed when assessing mesothelial cell connectivity.

Parallel studies at 24 h post introduced mechanical peritoneal wall damage highlighted the persistence of this tissue insult that was beyond the ability of HWCO_2_ to ameliorate. By day 10, the effects of open surgery on peritoneal cellular architecture are no longer distinguishable from non-surgery mice but this was not the case where mechanical damage was introduced. As we observed previously HWCO_2_ during the 1 h of open-surgery led to substantial restoration of peritoneal integrity. These data replicate our previous experience where active airflow was used [5]. The mechanism(s) that are at work to accelerate repair or reduce the impact of damage post-surgery time remain unresolved. One possibility we have considered is that the increased perfusion of gases in the whole animal may influence this tissue repair. This is an area of ongoing research. Nevertheless, these comparative studies reinforce the observation that HWCO_2_ both reduces immediate tissue damage associated with desiccation as well as positively influencing tissue repair in the case were damage is actively introduced.

Having established a robust and reproducible platform for exploring the effects of different surgery modalities, we then addressed an outstanding surgery question in the context of desmoidogenesis. The unresolved issue of the role of abdominal surgical trauma associated with prophylactic colectomy as It relates to subsequent desmoidogenesis in familial adenomatous polyposis (FAP), is difficult to model and the effects of different surgery, complex to measure. Here, we developed a mouse model, which recapitulates that of FAP patients who are predisposed to desmoid tumors. Desmoidogenesis had been explored previously using a different *Apc* mutation (*Apc1638N*) that is associated with a longer latency to illness that *Apc^Min/^*^+^ mice and where the crossing with *p53* KO mice further accelerated tumor formation and particularly burden [9].

There is an established risk of desmoids in FAP [11]. Conversely, there is growing evidence that laparoscopic surgery is less prone to desmoidogenesis in FAP patients [12, 14]. As we explored multiple surgery approaches in mice predisposed to desmoids the sample size in each cohort of mice was small. Nevertheless, the mouse model we employed allowed the investigation of surgical intervention (open and laparoscopic) and desmoidogenesis whereby as in patients with FAP there was a significant positive correlation between time from surgery and increased desmoid tumor burden.

In conclusion, the use of mice provides an opportunity to explore the effects of different modes of surgery along with the role of different kinds of CO_2_. With regard to peritoneal damage, open surgery under conditions that recapitulate the airflow indicative of a modern operating theatre generates substantive damage. HWCO_2_ perfused into the abdomen of mice under such operating conditions significantly reduced tissue damage. Perhaps more interesting, HWCO_2_ use at the time of additional, focused and simulated surgical-induced damage did not affect the burden of damage 1 day later but had a substantive effect at day 10. The mechanism that underpins this effect is unclear.

The development of an operating platform, which recapitulates that used for patients, allowed the investigation of an alleged surgery-associated tumor model that is desmoidogenesis. The association was replicated and no association with CO_2_ type was evident nor did HWCO_2_ provide any substantive protection. Overall, these model platforms for investigating surgery modalities under tightly controlled conditions using genetically defined and well mice inform the design of studies in larger mammals and patients.
